# Survival and neurologic outcomes following aortic occlusion for trauma and hemorrhagic shock in a hybrid operating room

**DOI:** 10.1186/s13017-023-00484-w

**Published:** 2023-03-23

**Authors:** Jeremy A. Balch, Tyler J. Loftus, Philip A. Efron, Alicia M. Mohr, Gilbert R. Upchurch, R. Stephen Smith

**Affiliations:** Department of Surgery, University of Florida Health, PO Box 100108, Gainesville, FL 32610-0108 USA

## Abstract

**Background:**

Outcomes following aortic occlusion for trauma and hemorrhagic shock are poor, leading some to question the clinical utility of aortic occlusion in this setting. This study evaluates neurologically intact survival following resuscitative endovascular balloon occlusion of the aorta (REBOA) versus resuscitative thoracotomy at a center with a dedicated trauma hybrid operating room with angiographic capabilities.

**Methods:**

This retrospective cohort analysis compared patients who underwent zone 1 aortic occlusion via resuscitative thoracotomy (*n* = 13) versus REBOA (*n* = 13) for blunt or non-thoracic, penetrating trauma and refractory hemorrhagic shock (systolic blood pressure less than 90 mmHg despite volume resuscitation) at a level 1 trauma center with a dedicated trauma hybrid operating room. The primary outcome was survival to hospital discharge. The secondary outcome was neurologic status at hospital discharge, assessed by Glasgow Coma Scale (GCS) scores.

**Results:**

Overall median age was 40 years, 27% had penetrating injuries, and 23% had pre-hospital closed-chest cardiopulmonary resuscitation. In both cohorts, median injury severity scores and head-abbreviated injury scores were 26 and 2, respectively. The resuscitative thoracotomy cohort had lower systolic blood pressure on arrival (0 [0–75] vs. 76 [65–99], *p* = 0.009). Hemorrhage control (systolic blood pressure 100 mmHg without ongoing vasopressor or transfusion requirements) was obtained in 77% of all REBOA cases and 8% of all resuscitative thoracotomy cases (*p* = 0.001). Survival to hospital discharge was greater in the REBOA cohort (54% vs. 8%, *p* = 0.030), as was discharge with GCS 15 (46% vs. 0%, *p* = 0.015).

**Conclusions:**

Among patients undergoing aortic occlusion for blunt or non-thoracic, penetrating trauma and refractory hemorrhagic shock at a center with a dedicated, trauma hybrid operating room, nearly half of all patients managed with REBOA had neurologically intact survival. The high death rate in resuscitative thoracotomy and differences in patient cohorts limit direct comparison.

**Supplementary Information:**

The online version contains supplementary material available at 10.1186/s13017-023-00484-w.

## Introduction

Hemorrhage accounts for approximately 40% of all trauma-related deaths overall and more than 80% of all trauma deaths that occur in operating room [1–3]. Early hemorrhage control can mitigate the most common cause of potentially preventable traumatic death [4].

The optimal approach to hemorrhage control remains controversial and is influenced by mechanism and anatomic region of injury. For penetrating trauma, resuscitative thoracotomy has an approximately 15% overall survival, though for blunt and non-thoracic penetrating trauma, outcomes are worse, with in-hospital mortality for blunt trauma and cardiopulmonary resuscitation (CPR) greater than 5 min approaching 100% [5, 6]. Alternatively, resuscitative endovascular balloon occlusion of the aorta (REBOA) may control sub-diaphragmatic arterial hemorrhage and restore blood flow to the heart and brain.

In a sentinel comparison between resuscitative thoracotomy and REBOA among 285 patients in the prospective, multi-center Aortic Occlusion in Resuscitation for Trauma and Acute Care Surgery (AORTA) registry, overall survival to discharge was 2.5% for resuscitative thoracotomy and 9.6% for REBOA; median discharge Glasgow Coma Scale (GCS) scores among survivors were 3 for resuscitative thoracotomy and 9 for REBOA [7]. Resuscitative thoracotomy and REBOA cohorts had different baseline characteristics, hindering direct comparisons and suggesting that patient factors influenced surgical decision-making for aortic occlusion procedures. Regardless, outcomes for aortic occlusion in hemorrhagic shock due to blunt or non-thoracic, penetrating trauma are poor, and some question its clinical utility [8].

The authors observed greater survival and discharge GCS in a small, mixed population of trauma and non-trauma patients undergoing REBOA at their center [9]. Notably, the authors’ level 1 trauma center features a dedicated, trauma hybrid operating room with uniplanar angiographic capabilities; these technologies could affect the technical performance, timing, and efficacy of REBOA balloon positioning and other angiographic hemorrhage control procedures [10–12]. The study adds to the literature by assessing overall outcomes for REBOA and comparing outcomes between resuscitative thoracotomy versus REBOA for refractory hemorrhagic shock due to blunt or non-penetrating, thoracic injury at a level 1 trauma center featuring a dedicated, trauma hybrid operating room. This study tested the hypothesis that REBOA would be associated with greater incidence of neurologically intact survival.

## Methods

### Study population

This retrospective cohort analysis included 26 consecutive adult trauma patients with blunt or non-thoracic, penetrating injuries and refractory hemorrhagic shock managed at a level 1 trauma center with a dedicated trauma hybrid operating room. Derivation of the study population is illustrated in Additional file 1: Fig. S1. There were 1527 level 1 (highest acuity) trauma team activations during a 52-month period ending November 2019. These cases were identified in a prospective, institutional trauma registry.

Twenty-six adult (age 18 years or greater) patients with blunt or non-thoracic, penetrating trauma that underwent zone 1 aortic occlusion for refractory hemorrhagic shock, defined as systolic blood pressure less than 90 mmHg with a transient or no response to volume resuscitation. Aortic occlusion was performed by resuscitative emergency department anterolateral or clamshell thoracotomy and aortic cross-clamping in 13 patients and by REBOA in 13 patients. Cases of penetrating, thoracic injuries with hemorrhagic shock are a contraindication for REBOA and were therefore excluded. In these patients, thoracotomy alone provides direct exposure for operative control of exsanguinating hemorrhage. Institutional review board approval was obtained.

### Primary outcome and power analysis

The primary outcome was survival to hospital discharge. A power analysis was performed to estimate the number of subjects per cohort that would be necessary to detect a statistically significant difference in survival to hospital discharge. Because the availability of a dedicated trauma hybrid operating room could affect the technical performance, timing, and efficacy of REBOA balloon positioning and other angiographic hemorrhage control procedures, the power analysis was performed using data from the authors’ institution. In a prior study of REBOA use in a mixed population of trauma and non-trauma patients from the author’s institution, 30-day survival was 38%; survival among eight patients undergoing resuscitative thoracotomy during the same period was 0% [9]. During internal quality control audits of the REBOA experience at the authors’ institution, it was noted that survival to discharge had reached 54%. Using these proportions and setting *α* and *β* to conventional values of 0.05 and 0.80, respectively, the present study would be adequately powered to detect a statistically significant difference in survival to hospital discharge with 13 patients in each cohort [13].

### Secondary outcome and data collection

The secondary outcome was discharge GCS among survivors. Discharge GCS and other variables that were not available within the authors’ prospective institutional trauma registry were obtained by manual review of the electronic health records. Variables describing patient characteristics are shown in Table 1**.** Variables describing patient management are shown in Table 2. Timing of hemorrhage control was defined as attaining systolic blood pressure 100 mmHg or greater without ongoing vasopressor or blood product transfusion requirements or subsequent episodes of hypotension with systolic blood pressure less than 90 mmHg, consistent with consensus recommendations regarding blood pressure targets in damage control resuscitation after trauma [14]. Variables describing resuscitation included tranexamic acid administration within four hours and transfusion of red blood cells and plasma within 24 h. Additional, tertiary outcomes included survival past the emergency department, survival past the operating room, lengths of stay in the hospital and ICU, days on mechanical ventilation, discharge disposition, and complications classified by the Clavien–Dindo system that was adapted for trauma by Naumann et al. [15].

**Table 1:** Characteristics of blunt or non-thoracic, penetrating trauma patients undergoing zone 1 aortic occlusion via resuscitative thoracotomy versus resuscitative endovascular balloon occlusion of the aorta (REBOA)

Patient characteristics	All patients (*n* = 26)	Resuscitative thoracotomy (*n* = 13)	REBOA (*n* = 13)	*p*
Age	40 [27–63]	37 [22–45]	58 [31–68]	0.064
Female	6 (23%)	2 (15%)	4 (31%)	0.645
Injury severity score	26 [22–37]	26 [23–31]	26 [20–38]	0.877
Head-abbreviated injury score	2 [0–3]	2 [0–3]	2 [0–3]	0.831
Blunt injury	19 (73%)	9 (69%)	10 (77%)	> 0.999
Non-thoracic, penetrating injury	7 (27%)	4 (31%)	3 (23%)	> 0.999
Pre-hospital traumatic arrest	6 (23%)	5 (38%)	1 (8%)	0.160
Received pre-hospital CPR	5 (19%)	4 (31%)	1 (8%)	0.322
Duration of CPR (min)	10 [3–15]	10 [3–12]	3	–
Interval: injury to arrival (min)	45 [30–60]	47 [33–55]	34 [30–72]	0.537
Glasgow Coma Scale on arrival	3 [3–3]	3 [3–3]	3 [3–15]	0.015
Best eye-opening response	1 [1–1]	1 [1–1]	1 [1–4]	0.015
Best verbal response	1 [1–1]	1 [1–1]	1 [1–5]	0.015
Best motor response	1 [1–1]	1 [1–1]	1 [1–6]	0.015
SBP on arrival (mmHg)	70 [0–84]	0 [0–75]	76 [65–99]	0.009
Temperature (Celsius)	35.1 [34.4–35.9]	35.0 [33.2–35.9]	35.3 [34.9–36.0]	0.199
FAST performed	23 (88%)	10 (77%)	13 (100%)	0.220
FAST negative	7 (27%)	4 (31%)	3 (23%)	> 0.999
FAST equivocal	3 (12%)	2 (15%)	1 (8%)	> 0.999
FAST positive	13 (50%)	4 (31%)	9 (69%)	0.115
pH	7.15 [7.06–7.25]	7.08 [6.99–7.16]	7.19 [7.11–7.29]	0.067
Lactic acid (mmol/L)	7.4 [3.7–10.0]	8.2 [5.0–13.9]	4.0 [3.2–9.5]	0.056
Hemoglobin (g/dL)	10.2 [8.9–11.9]	9.4 [7.5–10.7]	10.5 [9.0–12.5]	0.192
International normalized ratio	1.3 [1.2–1.7]	1.4 [1.2–2.8]	1.3 [1.2–1.5]	0.755
Had a rapid TEG	12 (46%)	5 (38%)	7 (54%)	0.695
Activating clotting time (sec)	117 [105–146]	128 [113–207]	105 [97–121]	0.086
Alpha angle (degrees)	66.0 [45.0–69.6]	60.2 [32.6–66.0]	69.1 [46.9–75.3]	0.061
* K* time (min)	2.7 [1.8–4.7]	2.7 [2.3–5.6]	2.7 [1.8–4.7]	0.776
Maximum amplitude (mm)	50.4 [41.6–55.0]	44.1 [26.0–54.5]	53.3 [42.7–56.1]	0.372
Estimated percent lysis	0.1 [0.0–1.4]	1.2 [0.3–91.6]	0.0 [0.0–0.1]	0.076
Had a regular TEG	16 (62%)	6 (46%)	10 (77%)	0.226
Reaction time (min)	4.5 [3.4–6.9]	6.2 [3.1–9.2]	3.9 [3.2–5.2]	0.247
Alpha angle (degrees)	53.9 [44.0–71.5]	56.2 [26.5–72.8]	53.9 [45.9–71.0]	0.920
* K* time (min)	3.0 [1.3–4.9]	2.1 [1.3–5.2]	3.3 [1.3–5.2]	0.609
Maximum amplitude (mm)	51.4 [40.4–60.2]	52.0 [34.4–61.3]	51.4 [40.6–60.9]	0.688

*CPR*, closed-chest cardiopulmonary resuscitation; *SBP*, systolic blood pressure; *FAST*, focused assessment with sonography for trauma; *TEG*, thromboelastograph. Data are presented as median [interquartile range] or *n* (%)

**Table 2:** Hemorrhage control procedures and resuscitation parameters for blunt or non-thoracic, penetrating trauma patients undergoing zone 1 aortic occlusion via resuscitative thoracotomy versus resuscitative endovascular balloon occlusion of the aorta (REBOA)

Hemorrhage control and resuscitation	All patients (*n* = 26)	Resuscitative thoracotomy (*n* = 13)	REBOA (*n* = 13)	*p*
Initial aortic occlusion in ED	13 (50%)	11 (85%)	2 (15%)	< 0.001
Initial aortic occlusion in OR	13 (50%)	2 (15%)	11 (85%)	< 0.001
Other hemorrhage control procedures				
Heart laceration repair	2 (8%)	2(15%)	0 (0%)	0.480
Pulmonary resection	1 (4%)	1 (8%)	0 (0%)	> 0.999
Lung laceration repair	3 (12%)	2 (15%)	1 (8%)	> 0.999
Solid organ resection	8 (31%)	2 (15%)	6 (46%)	0.202
Solid organ repair	5 (19%)	2 (15%)	3 (23%)	> 0.999
Vascular bypass	1 (4%)	1 (8%)	0 (0%)	> 0.999
Primary repair of a named vessel	6 (23%)	2 (15%)	4 (31%)	0.645
Ligation of a named vessel	4 (15%)	1 (8%)	3 (23%)	0.593
Therapeutic angiography^a^	4 (15%)	0 (0%)	4 (31%)	0.096
Other adjunctive procedures				
Hollow viscous resection	2 (8%)	0 (0%)	2 (15%)	0.480
Hollow viscous repair	2 (8%)	1 (8%)	1 (8%)	> 0.999
Diaphragm repair	1 (4%)	0 (0%)	1 (8%)	> 0.999
CT or vascular surgery consultation	1 (4%)	1 (8%)	0 (0%)	> 0.999
Interventional radiology consultation	4 (15%)	0 (0%)	4 (31%)	0.096
Obtained hemorrhage control in OR^b^	11 (42%)	1 (8%)	10 (77%)	0.001
Arrival to hemorrhage control (min)	111 [62–154]	62	112 [93–157]	0.343
OR start to hemorrhage control (min)	58 [32–91]	32	60 [33–103]	0.342
TXA administered 0–4 h after arrival	9 (35%)	5 (38%)	4 (31%)	> 0.999
RBC transfusions 0–4 h after arrival	7.0 [4.0–14.8]	7.0 [5.5–13.5]	5.0 [4.0–17.5]	0.680
Plasma transfusions 0–4 h after arrival	6.0 [3.8–15.0]	7.0 [4.0–15.0]	4.0 [3.5–15.0]	0.718
RBC transfusions 4–24 h after arrival	2.0 [0.0–5.0]	0.0 [0.0–5.5]	2.0 [0.5–5.0]	0.397
Plasma transfusions 4–24 h after arrival	1.5 [0.0–5.0]	0.0 [0.0–4.5]	2.0 [0.0–5.5]	0.465

*ED*, emergency department, *REBOA*, resuscitative endovascular balloon occlusion of the aorta, *CT*, presented as n (%) or median [interquartile range]. Cardiothoracic, *IR*, Interventional Radiology, *TXA*, tranexamic acid, *RBC*, red blood cell. Data are presented as n (%) or median [interquartile range]

^a^Endovascular stent placement, balloon angioplasty, coil placement, or embolization. ^b^Systolic blood pressure 100 mmHg or greater without ongoing vasopressor or blood product transfusion requirements or subsequent episodes of hypotension with systolic blood pressure less than 90 mmHg

### Trauma hybrid operating room, REBOA equipment, technique, and staff training

Our institution built a dedicated trauma hybrid operating room in a repurposed and remodeled angiography suite on the second floor of the hospital directly above the emergency department. The hybrid operating room contains a ceiling-mounted C-arm and a fluoroscopy-compatible table with tilt functions. The hybrid operating room is immediately adjacent to a fluoroscopy control room.

According to institutional protocols, REBOA catheters were placed by trauma surgeons for patients with blunt or non-thoracic penetrating trauma, hemorrhagic shock (i.e., systolic blood pressure < 90 mmHg), and a transient response or no response to volume resuscitation [9]. The REBOA procedure was either initiated in the emergency department for some patients or in the operating room for others, depending on their clinical trajectory and hemodynamic response to resuscitation. Balloon inflation was initially performed in zone 1 for all patients. Subsequent balloon deflation and relocation to zone 3 was at the discretion of the attending trauma surgeon based on hemodynamic response, completion of operative hemorrhage control techniques, and injury patterns. Initially, REBOA was performed with a 12-Fr introducer and aortic occlusion balloon designed by Cook Medical (Bloomington, Indiana). Subsequently, REBOA was performed using a 7-Fr introducer and aortic occlusion balloon designed by Prytime Medical (Boerne, Texas). Notably, both aortic occlusion balloon catheters must be deflated to obtain distal aortic blood flow around the balloon, in contrast to newer catheters that allow a more controlled amount of distal aortic blood flow through channels within the balloon. The senior author trained trauma surgeons and senior residents (i.e., PGY4 and PGY5 residents) in REBOA concepts and techniques with a series of 90-min audiovisual presentations followed by hands-on simulation training [9].

### Statistical analysis

To determine whether REBOA would be associated with a higher rate of neurologically intact survival, the primary outcome (hospital discharge alive) and secondary outcome (GCS at hospital discharge) were directly compared between resuscitative thoracotomy and REBOA cohorts. To understand whether potential differences in outcomes were attributable to baseline patient characteristics and other hemorrhage control and resuscitation parameters, these factors were also directly compared between resuscitative thoracotomy and REBOA cohorts. Continuous variables were compared by the nonparametric Kruskal–Wallis test and reported as median values with interquartile ranges. Binary variables were compared by Fisher’s Exact test and reported as raw numbers with percentages. Significance level was set at 5%.

## Results

### The resuscitative thoracotomy cohort had greater magnitude of shock

Patient characteristics are listed in Table 1. Overall median age was 40 years, 23% were females, 27% had penetrating injuries, and 23% had pre-hospital, closed-chest cardiopulmonary resuscitation of unknown duration. At our institution, resuscitation efforts are terminated after 15 min of unsuccessful CPR after penetrating trauma or 10 min of unsuccessful CPR after blunt trauma from time EMS-witnessed arrest and no evidence of cardiac activity on ultrasound in accordance with American College of Surgeons Committee on Trauma Guidelines [16]. Median injury severity scores and head-abbreviated injury scale scores were 26 and 2, respectively. The REBOA cohort had somewhat greater age compared with the resuscitative thoracotomy cohort, though the difference was not statistically significant (58 vs. 37 years, *p* = 0.064). The incidence of pre-hospital traumatic arrest was 38% in the resuscitative thoracotomy cohort and 8% in the REBOA cohort (p = 0.160). Although median GCS on arrival in the emergency department was 3 in both groups, the distribution of GCS scores in the REBOA cohort was greater (*p* = 0.015). The resuscitative thoracotomy cohort also had lower systolic blood pressure on arrival (0 [0–75] vs. 76 [65–99], *p* = 0.009) and greater magnitude of acidosis, though the differences were not statistically significant (pH: 7.08 vs. 7.19, *p* = 0.067; lactic acid: 8.2 vs. 4.0, *p* = 0.056). Hemoglobin values and coagulation parameters were similar between cohorts.

### The REBOA cohort had a greater incidence of hemorrhage control in the operating room

Hemorrhage control procedures and resuscitation parameters are listed in Table 2. Aortic occlusion was performed in the emergency department for 85% of all resuscitative thoracotomy cases and 15% of all REBOA cases (*p* < 0.001) and was performed in the operating room for the remaining cases. In the ED REBOA patients, there was no difference in time to hemorrhage control or patient outcomes from the overall REBOA cohort. There was a somewhat higher incidence of consultation with Interventional Radiology and therapeutic angiography performed in the REBOA cohort, though the differences were not statistically significant (31% vs. 0%, *p* = 0.096). The frequencies of other hemorrhage control and adjunctive operative procedures were similar between cohorts. The most commonly performed procedures in both cohorts were solid organ repair or ligation of a named vessel. Intraoperative consultation with cardiothoracic or vascular surgery was obtained in one resuscitative thoracotomy case (8%) and none of the REBOA cases. Hemorrhage control was obtained in 77% of all REBOA cases and 8% of all resuscitative thoracotomy cases (*p* = 0.001). The median time to hemorrhage control (SBP 100 with no ongoing vasopressor or transfusion requirements) in the REBOA cohort was 112 min after arrival and 60 min after operating room start time. Administration of tranexamic acid within four hours and blood products within 24 h was similar between cohorts.

### The REBOA cohort had greater incidence of neurologically intact survival

**Fig. 1:**
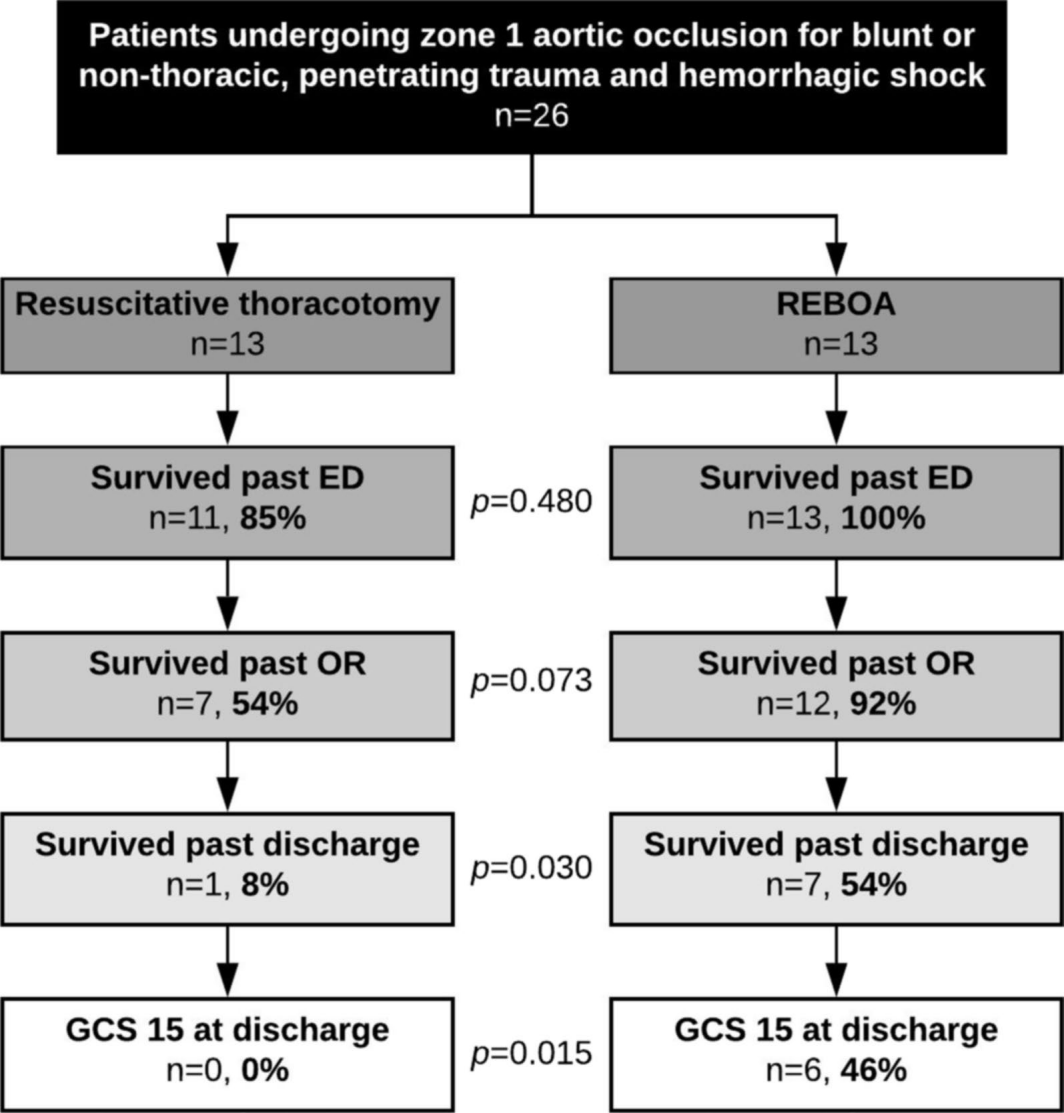
Forty-six percent of all patients undergoing REBOA for blunt or non-thoracic, penetrating trauma and hemorrhagic shock achieved survival to discharge with GCS 15

Survival through phases of hospital admission is illustrated in Fig. 1. Survival past the emergency department was similar between cohorts (resuscitative thoracotomy: 85%; REBOA: 100%). Survival past the operating room was somewhat greater in the REBOA cohort, though the difference was not statistically significant (92% vs. 54%, *p* = 0.073). Survival past discharge was significantly greater in the REBOA cohort (54% vs. 8%, *p* = 0.030), as was discharge with GCS 15 (46% vs. 0%, *p* = 0.015). Median GCS at discharge was also greater in the REBOA cohort when considering all patients (9 [3–15] vs. 3 [3], *p* = 0.029) and when considering only those patients that survived to discharge (15 [15] vs. 8, *p* = 0.046). Other clinical outcomes are listed in Table 3. In the REBOA cohort, three deaths occurred after the family decided to withdraw life support (type 5A complication) and three deaths occurred during active treatment (type 5B complication); in the resuscitative thoracotomy cohort, all twelve deaths occurred during active treatment (*p* = 0.001). One REBOA patient developed a common femoral artery pseudoaneurysm, which did not require procedural intervention. Three REBOA patients developed acute kidney injury, which may have been related to a combination of suprarenal balloon occlusion of the aorta and prolonged hemorrhagic shock. No other complications were directly attributable to REBOA. The REBOA cohort had longer lengths of stay in the hospital and ICU and had more days on mechanical ventilation, as expected given greater survival.

**Table 3:** Clinical outcomes for blunt or non-thoracic, penetrating trauma patients undergoing zone 1 aortic occlusion via resuscitative thoracotomy versus resuscitative endovascular balloon occlusion of the aorta (REBOA)

Clinical outcomes	All patients (*n* = 26)	Resuscitative thoracotomy (*n* = 13)	REBOA (*n* = 13)	*p*
Clavien–Dindo complication class^a^				
Overall class, median	5 [4, 5]	5 [5–5]	4 [2–5]	0.016
Grade 1, *n* (%)	1 (4%)	0 (0%)	1 (8%)	> 0.999
Grade 2, *n* (%)	1 (4%)	1 (8%)	0 (0%)	> 0.999
Grade 3a, *n* (%)	1 (4%)	0 (0%)	1 (8%)	> 0.999
Grade 3b, *n* (%)	0 (0%)	0 (0%)	0 (0%)	> 0.999
Grade 4a, *n* (%)	2 (8%)	0 (0%)	2 (15%)	0.480
Grade 4b, *n* (%)	1 (4%)	0 (0%)	1 (8%)	> 0.999
Grade 5a, *n* (%)	3 (12%)	0 (0%)	3 (23%)	0.220
Grade 5b, *n* (%)	15 (58%)	12 (92%)	3 (23%)	0.001
Hospital length of stay (*d*)	2.0 [1.0–15.5]	1.0 [1.0–2.0]	12.0 [2.5–19.0]	0.006
ICU length of stay (*d*)	2.0 [1.0–9.0]	1.0 [1.0–2.0]	6.0 [2.5–15.0]	0.007
ICU-free hospital days	0.0 [0.0–0.5]	0.0 [0.0–0.0]	0.0 [0.0–5.5]	0.103
Days on mechanical ventilation	2.0 [1.0–3.3]	1.0 [1.0–2.0]	2.0 [1.5–6.0]	0.044
Ventilator-free ICU days	0.0 [0.0–5.3]	0.0 [.0–0.0]	1.0 [0.0–7.5]	0.017
Discharge disposition				
Home	3 (12%)	0 (0%)	3 (23%)	0.220
Subacute/inpatient rehabilitation	2 (8%)	1 (8%)	1 (8%)	> 0.999
Long-term acute care	2 (8%)	0 (0%)	2 (15%)	0.480
Hospice	1 (4%)	0 (0%)	1 (8%)	> 0.999
In-hospital mortality	18 (69%)	12 (92%)	6 (46%)	0.030
GCS at discharge (overall)	3 [3–11]	3 [3–3]	9 [3–15]	0.029
GCS 15 at discharge (overall)	6 (23%)	0 (0%)	6 (46%)	0.015
GCS at discharge (survivors)	15 [11–15]	8	15 [15–15]	0.046
GCS 15 at discharge (survivors)	6 (75%)	0 (0%)	6 (86%)	0.250

*ED*, emergency department, *ICU*, intensive care unit, *GCS*, Glasgow Coma Scale. Data are presented as n (%) or median [interquartile range]

^a^Adapted for trauma by Naumann et al. [15]

## Discussion

In this study of patients with blunt or non-thoracic, penetrating trauma and refractory hemorrhagic shock, patients undergoing resuscitative thoracotomy had greater magnitude of shock and poor outcomes, and patients undergoing REBOA had a high incidence of neurologically intact survival.

Due to the greater magnitude of shock in the resuscitative thoracotomy group, the authors do not conclude that REBOA is superior to resuscitative thoracotomy. Differences in patient demographics between cohorts suggest that there was procedural selection bias, which may be appropriate in many cases. A greater magnitude of shock may imply pulseless femoral artery, where percutaneous femoral access is technically difficult and femoral artery cut-down may not be as expeditious as left anterolateral thoracotomy and aortic cross-clamping. Rather, the analysis suggests that patient factors contributed to surgical decision-making regarding the performance of aortic occlusion procedures, and that outcomes following REBOA were surprisingly good. More than three quarters of the REBOA cohort obtained hemorrhage control and hemodynamic stability in the operating room, and nearly half were discharged alive with GCS 15. In addition, only half of all in-hospital mortalities in the REBOA cohort occurred during active treatment. This may have important implications for family members and caregivers who would prefer the opportunity to visit their loved one prior to withdrawal of life support and those who would consider organ donation.

Previous studies have reported similar results for resuscitative thoracotomy, but less favorable results for REBOA [6, 7]. As experience with REBOA among institutions and individual providers increases, the authors expect that REBOA outcomes will improve over time; indeed, improved mortality rates have been demonstrated recently [17]. Institutional resources may also affect REBOA outcomes. One of the reasons for performing the present study was that the authors believe that a dedicated, trauma hybrid operating room with biplanar angiographic capabilities may affect the technical performance, timing, and efficacy of REBOA balloon positioning and other angiographic hemorrhage control procedures. In particular, the availability of a ceiling-mounted C-arm and fluoroscopy-compatible operating room table facilitates fluoroscopic assessment of balloon inflation and positioning as well as the performance of adjunctive endovascular stenting and embolization, without substantially interrupting the performance of operative hemorrhage control procedures. Adjunctive angiographic hemorrhage control procedures may offer performance advantages for several injury patterns, such as hemodynamically unstable pelvic fractures and some solid organ injuries [18–22]. In addition, certain patients with major, hepatic venous injuries may benefit from resuscitative endovascular balloon occlusion of the vena cava (REBOVC) and selective embolization of portal venous hemorrhage [19, 23–25]. Dedicated trauma hybrid operating rooms have been adopted in other countries, but are rare in the USA [10–12]. More commonly, trauma surgeons have access to hybrid operating rooms that are shared with interventional radiologists as well as cardiac and vascular surgeons, and these rooms may be occupied with a non-trauma case when they are needed for managing a trauma patient presenting in extremis.

Other observations from this study are consistent with previous work. Differences in baseline characteristics of patients undergoing resuscitative thoracotomy versus REBOA are similar to those reported in the AORTA registry [7]. Although hybrid operating room use has been associated with shorter intervals between arrival and the start time of interventions for hemorrhage control, time to hemorrhage control has not been previously reported [10, 11]. However, in a review regarding hemorrhage control after severe truncal injury, Holcomb discusses unpublished observations from the Pragmatic Randomized Optimal Platelet and Plasma Ratio (PROPPR) study, in which time to hemostasis after arrival in the operating room was approximately 67 min [26]. Notably, time to hemostasis was also variable among sites involved in the study and was independently associated with reduced 30-day mortality. Based on subsequent publications from the PROPPR group, anatomic hemostasis was determined by the surgeon’s assessment that bleeding within the surgical field was controlled and no further hemostatic interventions were anticipated [27]. This definition is different than the one used in the present study; however, it seems reasonable to conclude that time to hemorrhage control in our study was generally consistent with the experience of centers involved in the PROPPR study.

This study was limited by selection bias inherent to its retrospective design and lack of generalizability inherent to its single-institution design. The authors attempted to minimize selection bias by including all consecutive cases meeting inclusion criteria. It is possible to perform prospective investigations of trauma patients presenting in hemorrhagic shock, as demonstrated by Brenner et al. [7]; however, prospective enrollment was beyond the scope of this study. Although the single-institution design of this study limits generalizability, it also allows for a unique assessment of REBOA outcomes in the context of a dedicated, trauma hybrid operating room, which is novel in the USA; this assessment may have value for other centers that are considering the implementation of a trauma hybrid operating room. While the sample size was small, the power analysis suggested that this study was adequately powered to detect a statistically significant difference in the primary outcome. The authors again emphasize that differences in baseline patient characteristics between resuscitative thoracotomy and REBOA cohorts preclude the conclusion that REBOA is superior to resuscitative thoracotomy for zone 1 aortic occlusion in blunt or non-thoracic, penetrating trauma and refractory hemorrhagic shock. These findings support the hypothesis that patient factors contribute to surgical decision-making regarding the performance of resuscitative thoracotomy vs. REBOA, and that further investigation is necessary to establish consensus recommendations regarding aortic occlusion in this patient population. Similarly, we were unable to control for patients who presented in hemorrhagic shock who proceeded directly to operating room without resuscitative thoracotomy or REBOA. It is possible that the REBOA patients may have benefited from expeditious, open surgical hemorrhage control regardless of REBOA placement. Multivariate analysis and propensity matching may help address this question in future studies using much larger, federated datasets from multiple institutions [28].

## Conclusions

Among patients undergoing zone 1 aortic occlusion for blunt or non-thoracic, penetrating trauma and refractory hemorrhagic shock at a center with a dedicated, trauma hybrid operating room, nearly half of all patients managed with REBOA had neurologically intact survival. Differences in baseline characteristics of patients undergoing resuscitative thoracotomy versus REBOA support the hypothesis that patient factors contribute substantially to surgical decision-making regarding controversial hemorrhage control procedures for trauma patients presenting in extremis. Specifically, resuscitative thoracotomy may be favored for patients with greater magnitude of shock, more urgency for hemorrhage control, and pulseless femoral arteries that are difficult to cannulate for REBOA insertion. Further research is needed to determine the potential advantages in early hemorrhage control offered by a dedicated, trauma hybrid operating room.

## Supplementary Information

**Additional file 1**. Study Population Characteristics.

## Data Availability

Raw data may be made available upon request.
