# Supplementary figures and images for: Survival and neurologic outcomes following aortic occlusion for trauma and hemorrhagic shock in a hybrid operating room
Source: World J Emerg Surg. 2023 Mar 23;18:21. doi: 10.1186/s13017-023-00484-w (PMC10035182; doi:10.1186/s13017-023-00484-w)

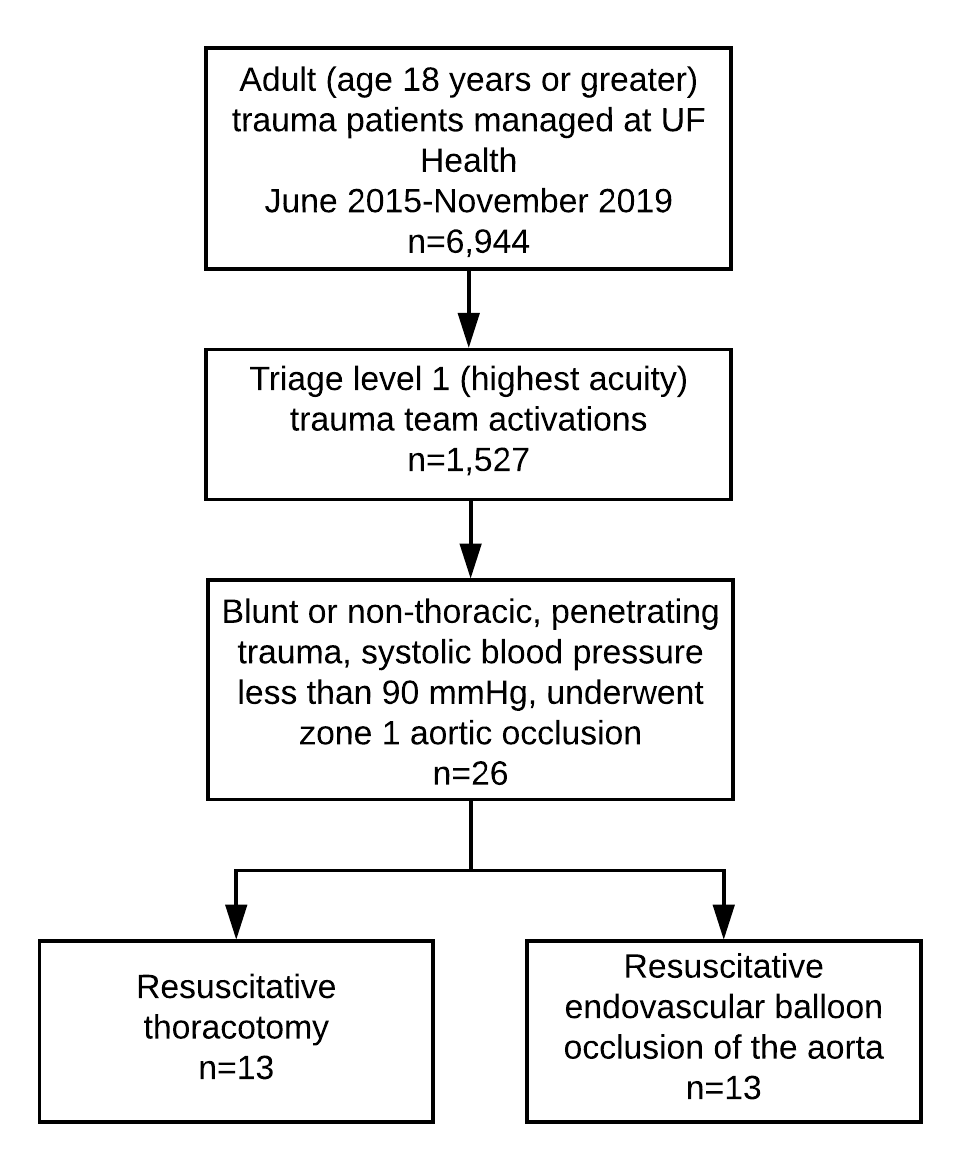

Supplement: Supplementary file 1 — Additional file 1. Study Population Characteristics. [file 13017_2023_484_MOESM1_ESM.tiff]
